# Regulation of Dendritic Synaptic Morphology and Transcription by the SRF Cofactor MKL/MRTF

**DOI:** 10.3389/fnmol.2021.767842

**Published:** 2021-11-02

**Authors:** Akiko Tabuchi, Daisuke Ihara

**Affiliations:** Laboratory of Molecular Neurobiology, Graduate School of Medicine and Pharmaceutical Sciences, University of Toyama, Toyama, Japan

**Keywords:** SRF, MKL, MRTF, gene expression, dendritic morphology, dendritic spine morphology, transcription

## Abstract

Accumulating evidence suggests that the serum response factor (SRF) cofactor megakaryoblastic leukemia (MKL)/myocardin-related transcription factor (MRTF) has critical roles in many physiological and pathological processes in various cell types. MKL/MRTF molecules comprise MKL1/MRTFA and MKL2/MRTFB, which possess actin-binding motifs at the N-terminus, and SRF-binding domains and a transcriptional activation domain (TAD) at the C-terminus. Several studies have reported that, in association with actin rearrangement, MKL/MRTF translocates from the cytoplasm to the nucleus, where it regulates SRF-mediated gene expression and controls cell motility. Therefore, it is important to elucidate the roles of MKL/MRTF in the nervous system with regard to its structural and functional regulation by extracellular stimuli. We demonstrated that MKL/MRTF is highly expressed in the brain, especially the synapses, and is involved in dendritic complexity and dendritic spine maturation. In addition to the positive regulation of dendritic complexity, we identified several MKL/MRTF isoforms that negatively regulate dendritic complexity in cortical neurons. We found that the MKL/MRTF isoforms were expressed differentially during brain development and the impacts of these isoforms on the immediate early genes including *Arc/Arg3.1*, were different. Here, we review the roles of MKL/MRTF in the nervous system, with a special focus on the MKL/MRTF-mediated fine-tuning of neuronal morphology and gene transcription. In the concluding remarks, we briefly discuss the future perspectives and the possible involvement of MKL/MRTF in neurological disorders such as schizophrenia and autism spectrum disorder.

## Introduction

Serum response factor (SRF) was identified as a transcription factor that binds to the serum response element (SRE) in an immediate early gene (IEG), c-*fos* (Norman et al., 1988). SRF recognizes and binds to the CC(A/T)_6_GG sequence (called the CArG box) located in a subset of cytoskeletal genes including ACTB (β-actin) as well as several IEGs such as c-*fos*, *egr1*, and activity-regulated cytoskeleton-associated protein (*Arc*)/*Arg*3.1 (Ramanan et al., 2005; Kawashima et al., 2009). SRF knockout (KO) in the mouse central nervous system (CNS) induced abnormalities of neuronal circuit formation (Knöll et al., 2006), neuronal migration (Alberti et al., 2005), activity-dependent gene expression (Ramanan et al., 2005), long-term potentiation (Ramanan et al., 2005), and long-term depression (Etkin et al., 2006), clearly demonstrating that SRF regulates neuronal morphology and neuronal activity-dependent transcription (Knöll and Nordheim, 2009).

Activation of SRF is triggered by an extracellular signal-regulated kinase (ERK)/mitogen-activated protein kinase (MAPK) and small G protein Rho-actin signaling cascades (Gineitis and Treisman, 2001). Although MAPK propagates the signals by phosphorylating SRF cofactor ternary complex factor (TCF) and thereby controls TCF/SRF complex-meditated transcription (Buchwalter et al., 2004), how Rho-actin signaling activates SRF-mediated transcription over a long duration remained poorly understood.

**Figure 1:**
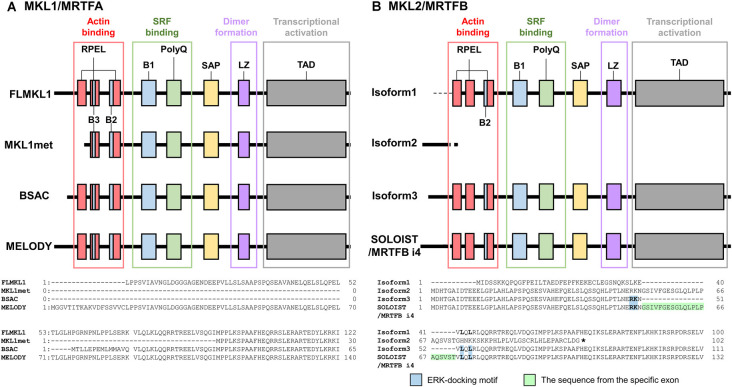
Structures of MKL/MRTF isoforms. MKL/MRTF contains RPEL motifs, a basic region (B1, B2, or B3), polyglutamine repeat (polyQ), SAF-A/B, Acinus, PIAS domain (SAP), leucine zipper domain (LZ), and transcriptional activation domain (TAD). Domains and their functions were illustrated using the article Kalita et al. (2012) as a reference. **(A)** Structures of rat MKL1/MRTFA isoforms and variation of the N-termini of isoforms modified from Ishikawa et al. (2013). Full-length MKL1 has two translational start sites, giving rise to FLMKL1 with three RPEL motifs and MKL1met with two RPEL motifs. FLMKL1, BSAC, and MELODY have individual 5′ exons and their N-terminal regions differ. **(B)** Structures of mouse MKL2/MRTFB isoforms and variation of the N-termini of isoforms modified from Ishibashi et al. (2020). Isoforms 1, 2, and 3 have individual 5′ exons, isoform 3 and SOLOIST/MRTFB i4 have the same 5′ exons. The difference between isoform 3 and SOLOIST/MRTFB i4 is a microexon encoding 21 amino acid residues, which disrupts the ERK-docking motif of isoform 3. SRF, serum response factor; MRTF, myocardin-related transcription factor; MKL, megakaryoblastic leukemia; BSAC, basic, SAP, and coiled-coil domain; MELODY, MKL1-elongated derivative of yield; FLMKL1, Full-length MKL1; SOLOIST, spliced neuronal long isoform of SRF transcriptional coactivator; RPEL, RPXXXEL; ERK, extracellular signal regulated kinase.

Another SRF cofactor, MKL/MRTF, links Rho-actin signaling to gene expression (Miralles et al., 2003; Posern and Treisman, 2006). MKL/MRTF molecules, MKL1/MRTFA [also termed megakaryocytic leukemia (MAL), basic, SAP, and coiled-coil domain (BSAC)] and MKL2/MRTFB (also termed MAL16), were identified (Sasazuki et al., 2002; Wang et al., 2002; Cen et al., 2003, 2004; Selvaraj and Prywes, 2003; Pipes et al., 2006) following the discovery of myocardin (Wang et al., 2001). MKL/MRTF proteins contain RPXXXEL (RPEL) motifs, basic domains (B), polyglutamine repeats (polyQ), SAF-A/B, Acinus, PIAS (SAP) domains, leucine zipper domains (LZ), and transcriptional activation domains (TAD; Miralles et al., 2003; Kalita et al., 2012; Figure 1). RPEL motifs bind to G-actins. B1 and polyQ are related to SRF binding, leucine zipper domains are involved in dimerization, and TAD is important for transcriptional activation (Miralles et al., 2003; Figure 1). MKL/MRTF binds to G-actins *via* RPEL motifs in the cytoplasm during the resting state of cells (Miralles et al., 2003). Once Rho is activated by extracellular stimuli, G-actin dissociates from MKL/MRTF to form F-actin, and MKL/MRTF translocates from the cytoplasm to the nucleus where it binds to and activates SRF (Miralles et al., 2003). The phosphorylation of MKL/MRTF by ERK/MAPK as well as F-actin formation is partly involved in its cellular localization and transcriptional activation (Panayiotou et al., 2016). This regulation of MKL/MRTF is thought to have key roles in many physiological and pathological processes (Miranda et al., 2021).

Neuronal plasticity, which is thought to be critical for learning and memory, is structurally and functionally regulated by extracellular stimuli. Because structural plasticity is associated with the actin cytoskeleton and functional plasticity is controlled by gene expression, it is important to elucidate the roles of MKL/MRTF in the brain. In this review, we introduce the expression pattern and localization of MKL/MRTF and then highlight neuronal morphology and transcription regulated by MKL/MRTF and its isoforms. In the concluding remarks, we will discuss the future perspectives and the possible relationship between the dysfunction of MKL/MRTF and neurological disorders.

## Expression and Localization of MKL/MRTF in The Brain and Neurons

Myocardin, the first MKL/MRTF family molecule identified, is highly expressed in the heart and smooth muscle (Wang et al., 2001). The mRNA expressions of MKL1/MRTFA and MKL2/MRTFB were originally measured by the whole mount *in situ* hybridization or northern blotting (Wang et al., 2002). To accurately compare the expression levels in tissues, we performed quantitative PCR and demonstrated that MKL1/MRTFA mRNA was highly expressed in the testis and brain and that MKL2/MRTFB mRNA was enriched in the brain (Ishikawa et al., 2010). Focusing on the prominent expression of MKL/MRTF in the brain, we performed *in situ* hybridization in the brain and found the hippocampus had the highest expression of MKL1/MRTFA followed by the cerebral cortex (Shiota et al., 2006). MKL2/MRTFB mRNA was highly expressed in the hippocampus, cortex, and striatum at the same level (Ishikawa et al., 2010). However, the cerebellum had a low mRNA level of MKL/MRTF (Shiota et al., 2006; Ishikawa et al., 2010). Furthermore, the expression of MKL/MRTF was upregulated during brain development. These findings suggest that MRTF might have functions in the forebrain.

Subsequently, we detected MRTF proteins in the brain and neurons. Immunostaining analysis demonstrated the hippocampus and cerebral cortex expressed MRTF proteins, with the prominent staining of cell bodies and dendrites in neurons (Shiota et al., 2006). To analyze the subcellular localization of MKL/MRTF in more detail, we generated and evaluated high quality antibodies against MKL1/MRTFA and MKL2/MRTFB (Kaneda et al., 2018). These antibodies specifically recognize the C-terminal regions of MKL1/MRTFA or MKL2/MRTFB and can be used for immunostaining, western blotting, and immunoprecipitation (Kaneda et al., 2018; Kikuchi et al., 2019). Using these antibodies, we successfully identified the synaptic localization of MKL1/MRTFA and MKL2/MRTFB: MRTF was concentrated in the postsynaptic density fraction, dendritic spines, and crude synaptic vesicle fractions (Kaneda et al., 2018).

## Regulation of Dendritic and Spine Morphology by MKL/MRTF

MRTF targets cytoskeletal genes, which prompted us to investigate the effect of MRTF on neuronal morphology. Interestingly, a series of mutants of MKL1/MRTFA uncovers the functional domains of MKL1/MRTFA, which were used to generate dominant negative mutants (Miralles et al., 2003). Because the basic 1 (B1) and basic 2 (B2) domains are required for the nuclear import of MKL1/MRTFA, the deletion of these two domains (ΔB1B2) inhibits the nuclear translocation of MKL/MRTF (Miralles et al., 2003). Deletion of the transcriptional activation domain (TAD) at the C-terminus (C471Δ) resulted in the lack of transcription (Miralles et al., 2003). We used these two dominant negative mutants, ΔB1B2 and C471Δ, to investigate the functional roles of MKL/MRTF in terms of SRF-mediated transcriptional responses and the dendritic complexity of cortical neurons. Overexpression of the ΔB1B2 or C471Δ mutant with an SRF-luciferase reporter gene revealed that the suppression of SRF-mediated transcriptional responses was associated with a reduction of dendritic complexity in cortical neurons (Shiota et al., 2006). Consistent with our observation, utilization of C471Δ in hippocampal neurons impeded neurite outgrowth and guidance (Knöll et al., 2006). Furthermore, to investigate the morphological roles of MRTF, we knocked down endogenous MKL/MRTF in cortical neurons by expressing small hairpin RNA (Ishikawa et al., 2010). A reduction of dendritic complexity and SRE-reporter activation by RNAi supported the findings when using the dominant negative mutants (Ishikawa et al., 2010). As a pharmacological approach, we used CCG-1423, a Rho signaling inhibitor that blocks SRF-mediated transcriptional responses, probably through inhibiting the nuclear translocation of MKL/MRTF (Hayashi et al., 2014). We found that CCG-1423 reduced the dendritic complexity of cortical neurons (Kikuchi et al., 2017). In addition to studies of primary cultured neurons *in vitro*, an *in vivo* study of brain-specific MRTF double KO mice demonstrated the importance of MRTF in neuronal morphology: the phenotype showed a defect in the arrangement of cortical and hippocampal neurons and a decrease in neuronal projection (Mokalled et al., 2010). The aberrant neuronal migration and impaired neurite outgrowth were observed in brain-specific MRTF double KO mice and the same phenotypes were also observed in brain-specific SRF KO (Mokalled et al., 2010). Taken together, these findings suggest that MRTF regulates neuronal morphology.

We found that MKL/MRTF is localized at postsynapses. What is the physiological meaning of this postsynaptic localization? To address this issue, we focused on dendritic spine morphology. The percentage of mature spines with a mushroom- or stubby-shaped structure was decreased when MKL1/MRTFA or MKL2/MRTFB was knocked down, suggesting MKL/MRTF promotes the maturation of dendritic spines (Kaneda et al., 2018). A similar dendritic spine morphology phenotype was observed in adult mouse SRF deletion *in vivo* (Nader et al., 2019).

A series of studies on SRF and its cofactors MRTF and TCF in neuronal morphology were already summarized in Table 1 of the review described by Knöll (2011). Here we update the information by adding the studies on dendritic spine maturation [MRTF knockdown experiments in cortical and hippocampal neurons (Kaneda et al., 2018) and adult mouse SRF deletion *in vivo* (Nader et al., 2019)] and the pharmacological study on dendritic complexity (application of CCG-1423 to cortical neurons; Kikuchi et al., 2017). These findings were already described above.

MKL/MRTF is a bifunctional protein that binds to G-actin, which may directly modulate neuronal morphology, and functions as a transcriptional coactivator in the nucleus. Therefore, it is unknown whether MKL/MRTF regulates dendritic or synaptic morphology *via* local and direct actions on the cytoskeleton, independent of gene expression, at dendrites and/or by moving from synapses into the nucleus and regulating the expression of IEGs and cytoskeletal genes.

## Differential Roles of MKL/MRTF Isoforms in Dendritic Morphology and Gene Expression

We identified several MKL1/MRTFA and MKL2/MRTFB isoforms and investigated their roles in the dendritic function and SRF-mediated gene expression.

We cloned rat MKL1/MRTFA isoforms, full-length MKL1 (FLMKL1), MKL1met, BSAC, and MKL1-elongated derivative of yield (MELODY; Ishikawa et al., 2013). Full-length MKL1, BSAC, and MELODY possess three RPEL motifs whereas MKL1met, which is produced by an alternative translational start site from FLMKL1, has two RPEL motifs (Ishikawa et al., 2013; Figure 1). Overexpression of MELODY, BSAC, or FLMKL1 in cortical neurons had no drastic impact on dendritic morphology or SRF-mediated transcription. However, MKL1met, which was localized to the nucleus more than the other isoforms, induced drastic SRF-mediated transcription and reduced dendritic complexity (Ishikawa et al., 2014). Therefore, the effect of MKL1met on dendritic morphology is associated with its nuclear localization and the strength of SRF-mediated gene expression.

**Figure 2:**
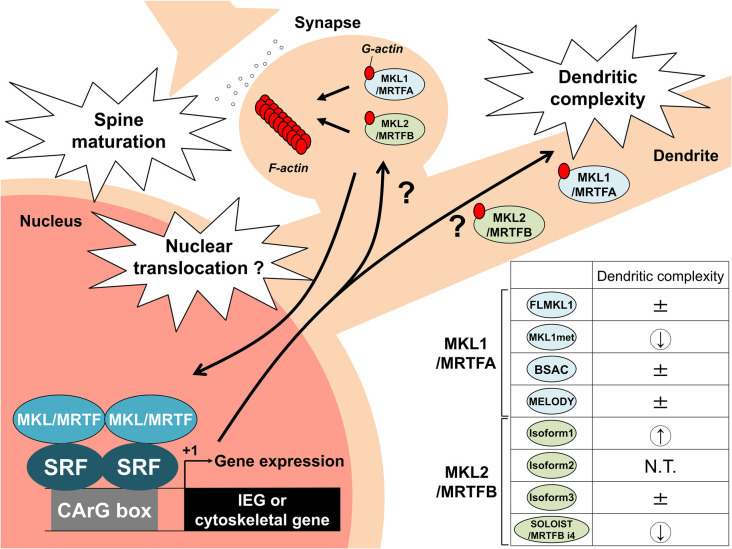
Schematic model by which MKL/MRTF and its isoforms regulate neuronal morphology and transcription. MKL/MRTF is localized in dendrites and dendritic spines. MKL/MRTF is an actin-binding protein and SRF cofactor. MKL/MRTF might act locally in dendrites and dendritic spines as well as bind to SRF to regulate IEGs or cytoskeletal genes in the nucleus to modulate spine maturation and dendritic complexity. In addition, the generation of multiple MKL/MRTF isoforms might fine-tune neuronal morphology and gene transcription in neurons. The overexpression studies of MKL/MRTF isoforms in terms of dendritic complexity are summarized on the right side. ± indicates no change; a down arrow indicates a decrease in dendritic complexity, and an up arrow indicates an increase in dendritic complexity. N.T. indicates not tested. IEG, immediate early gene.

In addition to MKL1/MRTFA, we cloned and analyzed several MKL2/MRTFB isoforms. Overexpression of MKL2/MRTFB isoform 1 in cortical neurons increased dendritic complexity, which was opposite to that of MKL1met (Ishikawa et al., 2010). These findings suggest that MKL1/MRTFA and MKL2/MRTFB might have their own target genes in the nucleus and/or their own target molecules at dendrites, reflecting different dendritic phenotypes. Recently, we also identified a novel and neuronal mouse MRTFB isoform named spliced neuronal long isoform of SRF transcriptional coactivator (SOLOIST)/MRTFB isoform 4 (MRTFB i4; Ishibashi et al., 2020). MKL2/MRTFB isoform 1 and SOLOIST/MRTFB i4 are enriched in neurons, but not in astrocytes and are upregulated during brain development. In terms of dendritic morphology, however, these two isoforms have opposite effects: the overexpression of isoform 1 increased dendritic complexity, whereas the overexpression of SOLOIST/MRTFB i4 decreased dendritic complexity (Ishibashi et al., 2020; Figure 1). SOLOIST/MRTFB i4 is a longer isoform with 21 amino acid residues ahead of the first RPEL motif in the MKL2/MRTFB isoform 3. MKL2/MRTFB isoform 3 has an ERK/MAPK-docking motif (RKNVLQL) that overlaps with the first RPEL. Interestingly, the ERK/MAPK-docking motif in SOLOIST/MRTFB i4 was disrupted by the additional 21 amino acid insertion (Ishibashi et al., 2020). Because the ERK/MAPK-docking motif in MKL1/MRTFA is required for its phosphorylation (Panayiotou et al., 2016), the unique amino acid residues of SOLOIST/MRTFB i4 might also affect its phosphorylation-dependent function, contributing to the negative regulation of dendritic complexity. We hypothesized that these isoforms have the opposite effect on dendritic complexity because of different IEGs or cytoskeletal gene induction. Therefore, we investigated the effect of MKL2/MRTFB isoforms on IEGs and cytoskeletal genes. Overexpression of these isoforms in Neuro-2a cells differentially increased endogenous IEG, c-*fos*, *egr*1, and *Arc/Arg*3.1 but did not markedly alter cytoskeletal *actinin* α1, *ACTB* (β-*actin*), *gelsolin*, and *srf* (Ishibashi et al., 2020). Although the strength of inducibility was different among isoforms (e.g., isoform 1 was the most effective), the levels of these gene inductions alone do not explain the different dendritic phenotypes. Other target genes or local functions at dendrites should be considered. We summarized the differential roles of the MRTF isoforms in Figure 2.

## Discussion

In this review, we summarized the functions of MKL/MRTF in neuronal morphology and transcription. In this section, we discuss where MKL/MRTF might regulate neuronal morphology. We demonstrated that MKL/MRTF, an actin-binding protein, is localized in dendrites and synapses, suggesting MKL/MRTF might regulate morphology locally by directly acting on the cytoskeleton without transcriptional coactivation. Interestingly, in addition to MKL/MRTF, SRF after nerve injury was also localized in the cytoplasm (Stern et al., 2013). The analysis of cytoplasmic SRF using green fluorescent protein (GFP)-fused SRF lacking a nuclear localization signal (SRF-ΔNLS-GFP) demonstrated the extranuclear roles of SRF, which were associated with the actin-severing factor cofilin, to regulate axonal outgrowth and branch formation (Stern et al., 2013). Furthermore, there is other evidence for a local role of MRTF and SRF in cilium turnover (Speight et al., 2021). Therefore, it would be interesting if MRTF interacts locally with SRF in the cytoplasm to regulate neuronal morphology. Further studies (e.g., the effect of RPEL motifs of MRTF on neuronal morphology) should be performed to obtain direct evidence about the local role of MRTF as an actin-binding protein in synapses and dendrites.

Next, we focused on the relationship between MRTF-target gene transcription and neuronal morphology using brain-specific MRTF double KO mice and the MRTF isoforms we identified. As described above, brain-specific MRTF double KO mice had abnormal brain structures (Mokalled et al., 2010). Mokalled et al. (2010) reported that MRTF regulated the representative cytoskeletal genes *ACTB* (β-actin), *gelsolin*, and the newly identified MRTF-target gene, *Pctaire*, which cooperatively acts with Cdk5 for cytoskeletal rearrangement, and thereby influences neurite outgrowth. Of note, the authors performed microarray analysis using MRTF brain-specific double KO mice and found MRTF might be involved in *Arc/Arg*3.1 gene induction *in vivo* (Mokalled et al., 2010). A study of the regulatory element of *Arc/Arg*3.1 identified an enhancer element named SARE, which consists of CREB, MEF2, and SRF-binding elements (Kawashima et al., 2009). Several studies, including ours, found that brain-derived neurotrophic factor (BDNF) strongly induced *Arc/Arg*3.1 gene transcription *via* an SRE within SARE (Pintchovski et al., 2009; Fukuchi et al., 2015). Thus, these findings prompted us to examine whether MKL/MRTF is involved in the BDNF-mediated activation of SARE in the *Arc/Arg*3.1 gene. Overexpression, RNAi-mediated knockdown, and chromatin immunoprecipitation (ChIP) analyses demonstrated the involvement of MKL2/MRTFB in the BDNF-induced activation of SARE in the *Arc/Arg*3.1 gene in cortical neurons although the involvement of MKL1/MRTFA might be very small, if any (Kikuchi et al., 2019). SARE is present in several neuronal genes as well as the *Arc/Arg*3.1 gene (Rodríguez-Tornos et al., 2013). Therefore, SARE-driven genes might be simultaneously activated by MKL2/MRTFB and may contribute to neuronal morphology.

Overexpression studies of MKL/MRTF isoforms showed that MKL1met decreased dendritic morphology, which was associated with more nuclear localization and had a greater effect in terms of SRF-mediated gene expression, compared with other MKL1/MRTFA isoforms (Ishikawa et al., 2014). However, the MKL2/MRTFB isoform 1 increased dendritic complexity (Ishikawa et al., 2010). These findings suggest that MKL1met-driven transcription is, at least in part, required for simplifying dendrites and the target gene differs from the MKL2/MRTFB target genes. A further complication is that opposite effects on dendritic complexity were observed for MKL2/MRTFB isoform 1 and SOLOIST/MRTFB i4 (Ishibashi et al., 2020). Overexpression studies of isoform 1 or SOLOIST/MRTFB i4 revealed that both isoforms activated IEG, c-*fos*, *egr*1, and *Arc/Arg3.1* although there was a difference in their ability to induce these genes. However, the effects on these genes do not explain the mechanism of opposite regulation related to dendritic complexity. As described above, in addition to their role in local dendrites, it is also necessary to consider the existence of unknown isoform-specific genes. Taken together, the MKL/MRTF isoforms might fine-tune gene transcription and dendritic morphology.

## Concluding Remarks

Synapse-to-nucleus and nucleus-to-synapse signaling are important neuronal plasticity-related events that reflect neuronal activity and regulate the correct expression level of functional proteins. Thus, it is important to investigate whether MKL/MRTF translocates from the cytoplasm into the nucleus of neurons. In non-neuronal cells, actin rearrangement and the phosphorylation of MKL/MRTF regulate its cellular localization (Miralles et al., 2003; Panayiotou et al., 2016). Many phosphorylation sites of MKL1/MRTFA have been identified (Panayiotou et al., 2016). The sites of the ERK/MAPK-mediated phosphorylation of MKL1/MRTFA determine its export from the nucleus or its import into the nucleus (Panayiotou et al., 2016). Recently, PKA-ERK/MAPK signaling in the striatum was reported to induce the nuclear localization and transcriptional activity of MKL2/MRTFB (Ariza et al., 2021). Interestingly, that study suggested a model whereby the MAPK-mediated phosphorylation of MKL2/MRTFB facilitated the interaction of MKL2/MRTFB with CREB-binding protein (CBP), an interaction that might control SRF-dependent gene expression in the striatum (Ariza et al., 2021). Although we showed that BDNF stimulated MKL2/MRTFB to activate SARE in the *Arc/Arg*3.1 gene in cortical neurons, the molecular mechanism involved remains to be determined. A previous study showed that MKL1/MRTFA/SRF-driven transcription activated by BDNF was dependent on the ERK/MAPK pathway, which caused the phosphorylation of MKL1/MRTFA (Kalita et al., 2006). Therefore, it might be interesting to investigate whether the phosphorylation of MKL2/MRTFB by ERK/MAPK is a molecular mechanism for the BDNF-mediated SARE activation of the *Arc/Arg*3.1 gene.

A series of brain-specific SRF KO mice revealed that SRF dysfunction might be involved in neuropathology. Interestingly, neuropathological phenotypes were observed in adult SRF deletion: hyperactivity syndrome in the dopamine system (Parkitna et al., 2010) and after traumatic brain injury (Förstner and Knöll, 2020), and epileptogenesis (Kuzniewska et al., 2016; Lösing et al., 2017). In contrast to the physiological function of the SRF cofactor MKL/MRTF, the dysfunction of MKL/MRTF might be involved in the etiology of neurological disorders. The analysis of large-scale schizophrenia cohort studies identified a genome-wide significant schizophrenia risk locus at 22q13.1 and demonstrated that seven single nucleotide polymorphisms (SNPs) of the *MKL1/MRTFA* gene had the most significant association with schizophrenia (Luo et al., 2015). Although these SNPs were found in the intron, not the coding region of the *MKL1/MRTFA* gene (Luo et al., 2015), they may affect the expression of the *MKL1/MRTFA* gene. Furthermore, a significant association of the *MKL2/MRTFB* gene with autism spectrum disorder (ASD) (Holt et al., 2010) and a *de novo* mutation of the *MKL2/MRTFB* gene, which caused an amino acid substitution in a patient with ASD (Neale et al., 2012), were reported. Therefore, it would be of interest to investigate whether SNPs and mutated MKL/MRTF genes affect the function of MKL/MRTF, leading to schizophrenia or ASD. To elucidate the molecular mechanism by which MKL/MRTF dysfunction is involved in such neurological disorders, the properties of MKL/MRTF described here (e.g., extranuclear, nuclear roles, protein modification in the regulation of subcellular localization and function) should be considered.

## Author Contributions

AT wrote the manuscript. DI assisted in making the figures. All authors edited and revised the manuscript and approved the final version of the manuscript for publication. All authors contributed to the article and approved the submitted version.

## Conflict of Interest

The authors declare that the research was conducted in the absence of any commercial or financial relationships that could be construed as a potential conflict of interest.

## Publisher’s Note

All claims expressed in this article are solely those of the authors and do not necessarily represent those of their affiliated organizations, or those of the publisher, the editors and the reviewers. Any product that may be evaluated in this article, or claim that may be made by its manufacturer, is not guaranteed or endorsed by the publisher.

## Abbreviations

ASD: autism spectrum disorder
Arc/Arg3.1: activity-regulated cytoskeleton-associated protein
BSAC: basic, SAP, and coiled-coil domain
ERK: extracellular signal-regulated kinase
IEG: immediate early gene
MAL: megakaryocytic acute leukemia
MELODY: MKL1-enlongated derivative of yield
MKL: megakaryoblastic leukemia
MRTF: myocardin-related transcription factor
RPXXXEL: arginine proline XXX glutamate leucine
SRF: serum response factor
SOLOIST: spliced neuronal long isoform of SRF transcriptional coactiavtor
TCF: ternary complex factor.
